# Molecular evidence of sequential evolution of DDT- and pyrethroid-resistant sodium channel in *Aedes aegypti*

**DOI:** 10.1371/journal.pntd.0007432

**Published:** 2019-06-03

**Authors:** Mengli Chen, Yuzhe Du, Shaoying Wu, Yoshiko Nomura, Guonian Zhu, Boris S. Zhorov, Ke Dong

**Affiliations:** 1 Institute of Pesticide and Environmental Toxicology, Zhejiang University, Hangzhou, China; 2 Department of Entomology, Genetics and Neuroscience Programs, Michigan State University, East Lansing, United States of America; 3 Department of Biochemistry and Biomedical Sciences, McMaster University, Hamilton, Ontario, Canada; 4 Sechenov Institute of Evolutionary Physiology & Biochemistry, Russian Academy of Sciences, St. Petersburg, Russia; Centers for Disease Control and Prevention, UNITED STATES

## Abstract

**Background:**

Multiple mutations in the voltage-gated sodium channel have been associated with knockdown resistance (kdr) to DDT and pyrethroid insecticides in a major human disease vector *Aedes aegypti*. One mutation, V1016G, confers sodium channel resistance to pyrethroids, but a different substitution in the same position V1016I alone had no effect. In pyrethroid-resistant *Ae*. *aegypti* populations, V1016I is often linked to another mutation, F1534C, which confers sodium channel resistance only to Type I pyrethroids including permethrin (PMT), but not to Type II pyrethroids including deltamethrin (DMT). Mosquitoes carrying both V1016G and F1534C exhibited a greater level of pyrethroid resistance than those carrying F1534C alone. More recently, a new mutation T1520I co-existing with F1534C was detected in India. However, whether V1016I or T1520I enhances pyrethroid resistance of sodium channels carrying F1534C remains unknown.

**Methodology/Principal findings:**

V1016I, V1016G, T1520I and F1534C substitutions were introduced alone and in various combinations into AaNa_v_1-1, a sodium channel from *Aedes aegypti*. The mutant channels were then expressed in *Xenopus* oocytes and examined for channel properties and sensitivity to pyrethroids using the two-electrode voltage clamping technique. The results showed that V1016I or T1520I alone did not alter the AaNa_v_1-1 sensitivity to PMT or DMT. However, the double mutant T^1520^I+F^1534^C was more resistant to PMT than F^1534^C, but remained sensitive to DMT. In contrast, the double mutant V^1016^I+F^1534^C was resistant to DMT and more resistant to PMT than F^1534^C. Furthermore, V^1016^I/G and F^1534^C channels, but not T^1520^I, were resistant to dichlorodiphenyltrichloroethane (DDT). Cryo-EM structures of sodium channels suggest that T1520I allosterically deforms geometry of the pyrethroid receptor site PyR1 in AaNa_v_1-1. The small deformation does not affect binding of DDT, PMT or DMT, but in combination with F1534C it increases the channel resistance to PMT and DDT.

**Conclusions/Significance:**

Our data corroborated the previously proposed sequential selection of kdr mutations in *Ae*. *aegypti*. We proposed that mutation F1534C first emerged in response to DDT/pyrethroids providing a platform for subsequent selection of mutations V1016I and T1520I that confer greater and broader spectrum of pyrethroid resistance.

## Author summary

Intensive use of pyrethroids has led to the selection of resistance in mosquitoes, and knockdown resistance (kdr) is one of the major mechanisms of pyrethroid resistance. So far, eleven kdr mutations were identified to be associated with pyrethroid resistance in *Aedes aegypti*. Among the mutations, the V1016I and T1520I substitutions were found to be associated with F1534C but rarely found alone. F1534C confers sodium channel resistance to Type I pyrethroids including permethrin (PMT). However, whether V1016I or T1520I enhances the F1534C-mediated sodium channel resistance remain unknown. In this study, our electrophysiological results confirmed their involvement in kdr and corroborate the previously proposed sequential selection of kdr mutations in *Ae*. *aegypti*: F1534C likely emerged first in response to DDT and/or pyrethroids, whereas V1016I and T1520I appeared later under more intensive selection from pyrethroid use.

## Introduction

Pyrethroid insecticides are synthetic analogs of naturally occurring pyrethrins from *Chrysanthemum* spp. [1]. Due to their low mammalian toxicity, high insecticidal activity and fast action, pyrethroids are currently a dominant class of insecticides used globally against mosquitoes and other human disease vectors. However, intensive use of pyrethroids has led to selection of resistant mosquitoes around the world. Pyrethroid resistance is currently a major obstacle in mosquito control [2].

**Fig 1:**
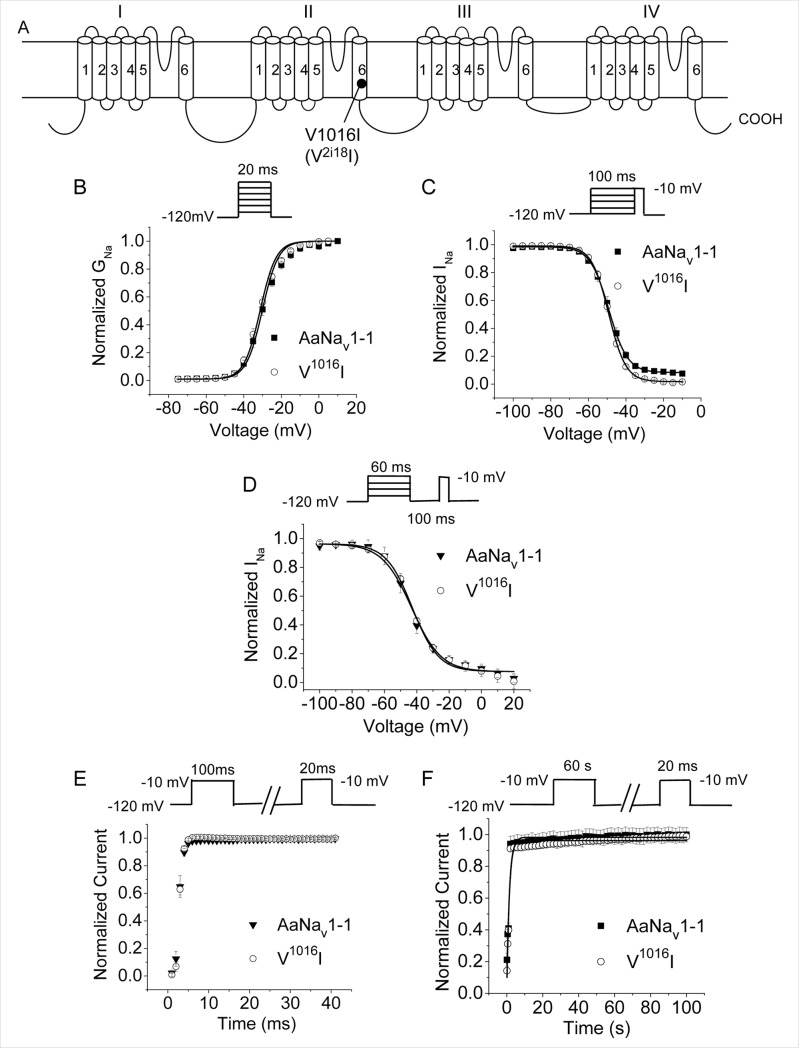
Gating properties of AaNa_v_1-1 and V1016I channels. (A) Membrane topology. (B) Voltage dependence of activation. (C) Voltage dependence of fast inactivation. (D) Voltage dependence of slow inactivation. (E) Recovery from fast inactivation. (F) Recovery from slow inactivation. Voltage step protocols used to generate the curves are shown above each panel.

**Fig 2:**
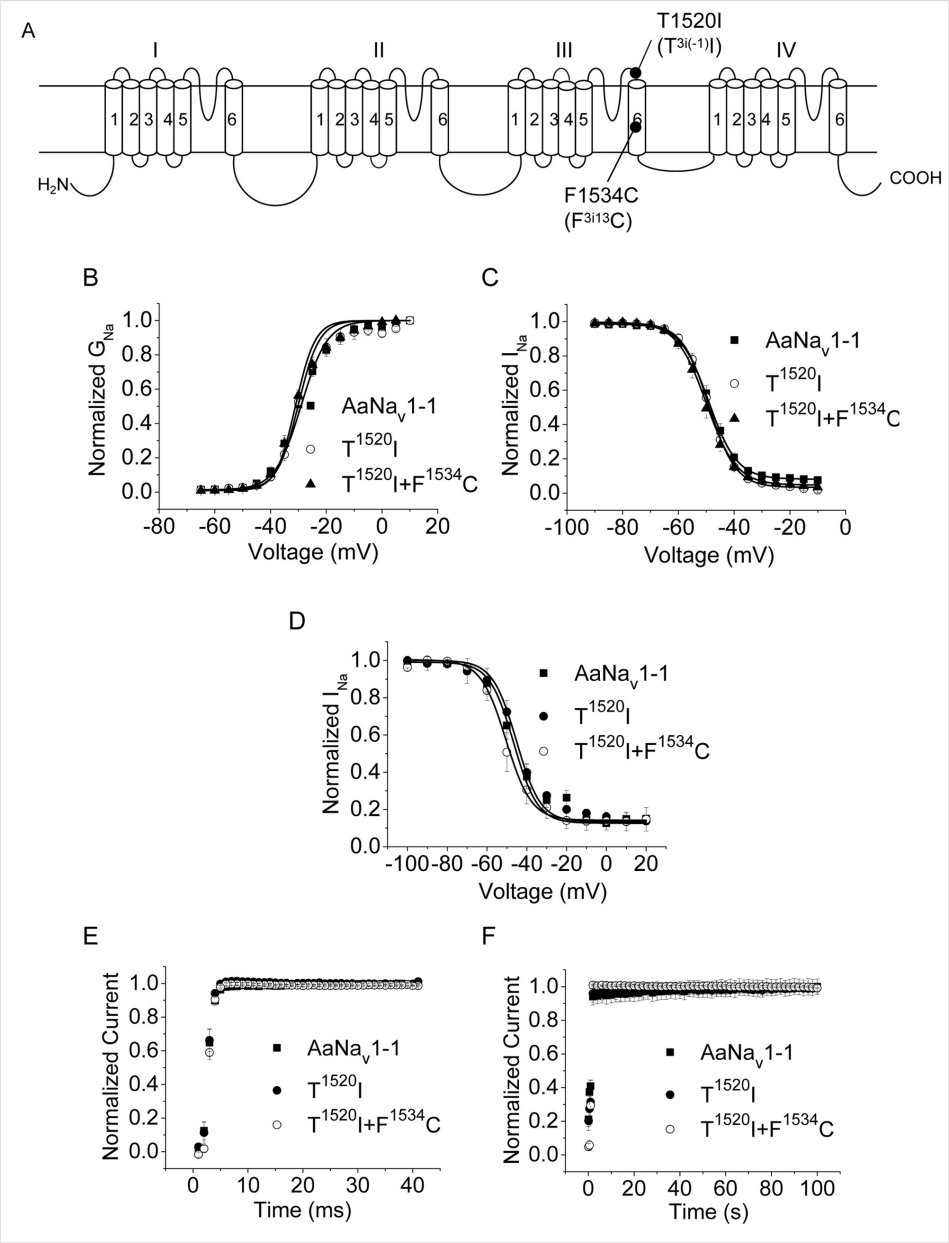
Gating properties of the AaNa_v_1-1, T1520I and T1520I/F1534C channels. (A) Positions of T1520I and F1534C. (B) Voltage dependence of activation. (C) Voltage dependence of fast inactivation. (D) Voltage dependence of slow inactivation. (E) Recovery from fast inactivation. (F) Recovery from slow inactivation. Voltage step protocols are the same as in Fig 1.

Pyrethroids target voltage-gated sodium channels in insects. The pore-forming α1 subunit of sodium channels has four homologous repeat domains (I-IV), each containing six transmembrane segments, S1-S6 (Figs 1A and 2A). In each repeat, segments S1-S4 constitute a voltage-sensing domain. Eight segments S5 and S6 along with four membrane-reentrant P-loops, which connect S5s and S6s, form the pore domain. Voltage-gated sodium channels are responsible for initiation and propagation of the action potential in almost all excitable cells [3]. In response to membrane depolarization, sodium channels open (activate) and allow sodium ions to flow into the cell, causing rapid membrane depolarization, the rising phase of action potentials. A few milliseconds after channel activation, sodium channels immediately undergo fast inactivation, which is critical for the action potential termination, preventing excessive membrane depolarization [4]. In response to prolonged depolarization (seconds to minutes) sodium channels progressively enter into more stable slow-inactivated states. Slow inactivation is important for regulation of membrane excitability, action potential firing patterns, and spike frequency adaptation [5].

Pyrethroids, as well as DDT which was used intensively before 1960s, prolong the opening of sodium channels by inhibiting their inactivation and deactivation [6–9]. Pyrethroids are categorized into two groups based on their poisoning symptoms, chemical structures and effects on sodium channels. Type II, but not Type I pyrethroids have an α-cyano group next to the phenylbenzylalcohol moiety. Type II pyrethroid, such as deltamethrin (DMT), inhibit deactivation of sodium channels to a greater extent than Type I pyrethroids, such as permethrin (PMT), inducing much slower decay of tail currents associated with repolarization. Our understanding of pyrethroid interaction with sodium channels at the molecular level began with identification of mutations that confer resistance to DDT and pyrethroids, known as knockdown resistance (kdr) mutations, in a wide variety of insect species including *Aedes aegypti*, a vector of viruses causing dengue fever, chikungunya, Zika fever, yellow fever and other diseases.

Eleven sodium channel mutations, V410L [10], G923V [11], L982W [11], S989P [12], V1016G/I [13–15], I1011M/V [16], T1520I [17], F1534C [15] and D1763Y [18] were found to be associated with pyrethroid resistance in *Ae*. *aegypti*. Co-occurrence of multiple mutations appears to be a common phenomenon in populations of pyrethroid-resistant *Ae*. *aegypti* [19]. Examples include V1016G/S989P [12, 20, 21], V1016G/F1534C [22] and V1016I/F1534C [14, 23]. Amino acid positions of these and other mutations in this study are numbered based on the house fly sodium channel protein (Genbank accession number: AAB47604).

Mutation V1016G located in IIS6 was first identified in PMT- and DDT-resistant *Ae*. *aegypti* from Indonesia, Vietnam and Thailand [11]. V1016G was often found associated with S989P in Thailand [12], Malaysia [21], Saudi Arabia [24] and other countries in the south-east Asia. S989P is located in the extracellular loop that connects segments IIS5 and IIS6. Another mutation in the same position, V1016I, was found in thirty *Ae*. *aegypti* populations in Latin America [25]. V1016I was always found co-existing with F1534C (segments IIIS6) in pyrethroid-resistant populations in South and North Americas [23, 26], Brazil [14, 27], Mexico [28, 29], and the USA [30]. In contrast, although co-existing mutations F1534C/V1016G were found in the DMT-treated *Ae*. *aegypti* populations in Singapore [22], they also occurred in separate haplotypic populations [31]. Interestingly, F1534C alone was found in many DDT- and PMT-resistant *Ae*. *aegypti* populations in Thailand, Vietnam [12] and Venezuela [23]. More recently, a new mutation T1520I at the extracellular N-end of IIIS6 was found to coexist with F1534C in India [17].

Previous studies demonstrated that mutation F1534C reduced sensitivity of the *Ae*. *aegypti* sodium channel, AaNa_v_1-1, and the cockroach sodium channel, BgNa_v_1-1a, to Type I pyrethroids (e.g., PMT and bioresmethrin) in the *Xenopus* oocyte expression system [32–34]. V1016G was likely selected under the pressure of pyrethroids because it conferred resistance to both Type I and Type II pyrethroids [32, 33]. However, whether V1016I or T1520I enhances the F1534C-mediated pyrethroid resistance remain unknown.

In this study, we introduced V1016I, F1534C and T1520I alone and in various combinations in the AaNa_v_1-1 channel, expressed the mutant channels in *Xenopus* oocytes, and examined their gating properties, pyrethroid and DDT sensitivity. We found that (i) like V1016I [26], T1520I did not alter the sensitivity of AaNa_v_1-1 channels to PMT or DMT, (ii) the T^1520^I+F^1534^C channel was resistant to PMT, but sensitive to DMT, (iii) the V^1016^I+F^1534^C channel was resistant to DMT and more resistant to PMT than the F^1534^C channel, and (iv) V^1016^I/G and F^1534^C channels, but not T^1520^I, were resistant to DDT.

Cryo-EM structures of sodium channels suggest that T1520I in AaNa_v_1-1 allosterically distorts the pyrethroid receptor site PyR1. The small distortion *per se* does not affect action of insecticides, which we studied here, but in combination with F1534C it affects binding of PMT and DDT.

Our results corroborated sequential selection of kdr mutations in *Ae*. *aegypti*: F1534C emerged first in response to DDT and/or pyrethroids, whereas V1016I and T1520I appeared later under more intensive selection from pyrethroid use.

## Materials and Methods

### Insecticides

(1R,3R,α-S)-deltamethrin and isomer-mixed cypermethrin were purchased from Sigma-Aldrich (Sigma-Aldrich, St. Louis, MO, USA). (1R)-cis-permethrin and bifenthrin were purchased from Chem Service (Chem Service, West Chester, PA, USA). β-cyfluthrin was purchased from Fluka (Fluka, Ronkonkoma, NY, USA). (1R)-cis-NRDC 157, which is structurally similar to deltamethrin, but lacks the α-cyano group next to the phenylbenzylalcohol moiety, was a gift from Bhupinder Khambay (Rothamsted Research, Harpenden, United Kingdom). The purities of these compounds were above 98%. Stock solutions of the compounds (100 mM) were dissolved in dimethyl sulfoxide (DMSO). The working solution was prepared in ND96 recording solution immediately prior to experiments. The concentration of DMSO in the final solution (< 0.5%) had no effect on the function of sodium channels in the experiments.

### Site-directed mutagenesis

V^1016^I, V^1016^G and F^1534^C channel constructs in the background of AaNa_v_1-1, a pyrethroid-sensitive sodium channel from *Ae*. *aegypti*, were available from a previous study [32]. In this study, we generated a double mutant V^1016^I+F^1534^C by introducing V1016I into the F^1534^C construct. We introduced T1520I into AaNa_v_1-1 or F^1534^C to generate T^1520^I and T^1520^I+F^1534^C mutant channels, respectively. Site-directed mutagenesis was performed by PCR using Phusion High-Fidelity DNA Polymerase (NEB, Ipswich, MA). The sequence of the primers used in mutagenesis to introduce T1520I mutation were “CAGCCGATTCGCGAGATCAACATCTACATGTACC” (forward primer) and “GGTACATGTAGATGTTGATCTCGCGAATCGGCTGC” (reverse primer). The mutant clones were verified by DNA sequencing.

### Expression of AaNa_v_1-1 sodium channel in Xenopus oocytes

AaNa_v_1-1 and mutants were expressed in the *Xenopus* oocytes, Ovaries from oocyte-positive female *Xenopus laevis* purchased from Xenopus 1 (Dexter, MI). The procedures for oocyte preparation, cRNA synthesis and injection were identical to those described previously [35]. cRNA was prepared by in vitro transcription with T7 polymerase using the mMESSAGE mMACHINE high yield capped RNA kit (Ambion, Austin, TX). To enhance expression of AaNa_v_1-1 and mutant channels, their cRNAs were co-injected into oocytes with *Ae*. *aegypti* tipE cRNA in the 1:1 molar ratio [36, 37].

### Electrophysiological recording and analysis

Sodium currents were recorded by using the oocyte clamp instrument OC-725C (Warner Instrument, Hamden, CT), Digidata 1200A, and pCLAMP 6 software interface (Axon Instruments Inc., Foster City, CA). Methods for electrophysiological recording and data analysis were similar to those described previously [38]. The peak current was recorded by -10 mV test pulse from a holding potential of -120 mV. The peak sodium current was limited to 2.0–3.0 μA to achieve better voltage control. This was achieved by adjusting the amount of cRNA and the incubation time after injection.

### Gating properties of sodium channels

The voltage dependence of sodium channel conductance (G) was calculated by measuring the peak current at test potentials ranging from -80 to +65 mV in 5 mV increments and divided by (V-V_rev_), where V is the test potential and V_rev_ is the reversal potential for sodium ions. The peak conductance values were normalized to the maximal peak conductance (G_max_) and fitted with a two-state Boltzmann equation:

$$G/G_{max}={\left[1+exp(V‐V_{1/2})/k\right]}^{-1}$$

where V_1/2_ is the voltage of half-maximal activation, and *k* is the slope factor.

The voltage dependence of sodium channel inactivation was determined by using 100 milliseconds prepulses ranging from -120 to -10 mV in 5 mV increments from a holding potential of -120 mV, followed by test pulses to -10 mV for 20 milliseconds. The peak current amplitude during the test depolarization was normalized to the maximal current amplitude and plotted as a function of the prepulse potential. Data were fitted with a two-state Boltzmann equation:

$$I/I_{max}={\left[1+(exp(V‐V_{1/2})/k)\right]}^{-1}$$

where I is the peak sodium current, I_max_ is the maximal current evoked, V is the potential of the voltage prepulse, V_1/2_ is the half maximal voltage for inactivation, and *k* is the slope factor.

Recovery time from fast inactivation was measured by a 100 milliseconds depolarizing pulse to -10 mV, then repolarization to -120 mV for an interval of variable duration, followed by a 20 milliseconds test pulse to -10 mV. The peak current during the test pulse was divided by the peak current during the inactivating pulse and plotted as a function of duration time between the two pulses. To determine the time constant for recovery, the curve was fitted by double exponential function:

$$I=1‐[A1\times exp\left(‐t/T_{1}\right)+A2\times exp\left(‐t/T_{2}\right)]$$

where A1 and A2 are the relative proportions of current recovering with time constants Ƭ_1_ and Ƭ_2_, and t is the recovery interval.

The voltage dependence of slow inactivation was measured with 60 milliseconds conditioning pulses ranging from -100 mV to 0 mV in 10 mV increments, followed by repolarization to a holding potential of -120 mV for 100 milliseconds to remove fast inactivation, and at last a -10 mV test pulse for 20 milliseconds. The peak current amplitude during the test depolarization was normalized to the maximal current amplitude and plotted against the pre-pulse potential. Data were fitted with a two-state Boltzmann equation as above for recovery from fast inactivation.

Recovery from slow inactivation was tested by a pre-pulse to -10 mV for 60 seconds to drive sodium channels into the slow inactivated state, followed by repolarization to -120 mV for 0 to 30 seconds, and finally a test pulse to -10 mV for 20 milliseconds. The peak current during the test pulse was divided by the peak current, which has a repolarizing duration of 30 seconds, and plotted as a function of duration between the prepulse and test pulses. Recovery from slow inactivation was fitted by a double exponential function as that used for recovery from fast inactivation.

### Measurement of tail currents induced by pyrethroids

The method of pyrethroids application in the recording system was identical to that described previously [35]. Effects were measured 10 minutes after the pyrethroids application. Insecticide-induced tail currents were recorded by using a 100-pulse train of 5 milliseconds step depolarization from -120 to 0 mV with 5 milliseconds inter-pulse intervals [39]. The percentage of sodium channels modified by pyrethroids was calculated using the following equation [40]:

$$M=\left\{\left[I_{tail}/\left(E_{h}‐E_{Na}\right)\right]/\left[I_{Na}/\left(E_{t}‐E_{Na}\right)\right]\right\}\times 100$$

where I_tail_ is the maximal tail current amplitude, E_h_ is the potential to which the membrane is repolarized, E_Na_ is the reversal potential for sodium currents determined from the current-voltage curve, I_Na_ is the amplitude of the peak current during depolarization before exposure to insecticides, and E_t_ is the potential of the step depolarization.

### Inhibition of inactivation induced by DDT

The inhibitory effect of DDT on the sodium channel inactivation was assayed by measuring the remaining current at the end of a 20 ms depolarization to -10 mV from a holding potential of -120 mV and normalizing it to the peak current. The DDT application and data analysis method were identical to those reported previously [41].

## Results

### Effects of V1016I and T1520I on the functional properties of AaNa_v_1-1

Sodium channels are critical for electrical signaling in the nervous system. Since kdr mutations could reduce insect fitness, we examined the functional properties of mutant channels V^1016^I, F^1534^C, T^1520^I, V^1016^I+F^1534^C and T^1520^I+F^1534^C expressed in *Xenopus* oocytes. All these channels generated sodium currents comparable to those in the wild-type channel. None of the mutants had any detectable changes in the voltage dependence of activation or fast inactivation (Table 1, Figs 1B, 1C, 2B and 2C). We further examined effects of mutations V1016I and T1520I on slow inactivation, and recovery from slow and fast inactivation. Neither V1016I nor T1520I significantly altered development of slow inactivation (Table 2, Figs 1D and 2D). The time courses of recovery from fast inactivation (Figs 1E and 2E) and slow inactivation (Figs 1F and 2F) of V^1016^I and T^1520^I channels were essentially the same as those of the AaNa_v_1-1 channel. Thus, neither V1016I nor T1520I modified any measured functional properties of AaNa_v_1-1 channel expressed in *Xenopus* oocytes.

**Table 1:** Voltage dependence of activation and fast inactivation of AaNa_v_1-1 and mutant sodium channels.

Na^+^ channel type	Activation		Inactivation		n
	V_1/2_ (mV)	*k* (mV)	V_1/2_ (mV)	*k* (mV)	
AaNa_v_1-1	-31.0 ± 0.6	5.7 ± 0.2	-50.2 ± 0.6	4.9 ± 0.3	8
V^1016^I	-30.3 ± 1.0	6.3 ± 0.2	-51.5 ± 0.8	4.6 ± 0.1	10
F^1534^C	-32.5 ± 0.5	4.1 ± 0.6	-50.6 ± 0.6	4.5 ± 0.1	8
V^1016^I+F^1534^C	-30.0 ± 0.6	4.6 ± 0.3	-47.2 ± 0.5	4.4 ± 0.1	9
T^1520^I	-31.0 ± 0.7	6.5 ± 0.6	-49.1 ± 0.8	4.6 ± 0.2	8
T^1520^I+F^1534^C	-30.9 ± 0.5	4.9 ± 0.3	-50.6 ± 0.9	4.6 ± 0.1	9

V_1/2_ is the voltage for half-maximal conductance or inactivation, *k* is the slope factor for conductance or inactivation.

The values in the table represent the mean ± s.e, and n is the number of oocytes used.

Statistical analysis was performed by one-way ANOVA with Scheffé's *post hoc* analysis (p < 0.05).

**Table 2:** Voltage dependence of slow inactivation of AaNa_v_1-1 and mutant sodium channels.

Na^+^ channel type	Slow inactivation		n
	V_1/2_ (mV)	*k* (mV)	
AaNa_v_1-1	-43.0 ± 2.3	8.6 ± 1.3	8
V^1016^I	-38.9 ± 1.1	11.1 ± 0.8	10
T^1520^I	-42.3 ± 1.7	6.0 ± 0.9	8
T^1520^I+F^1534^C	-48.2 ± 2.8	6.3 ± 0.3	7

V_1/2_ is the voltage for half-maximal inactivation, *k* is the slope factor for inactivation.

The values in the table represent the mean ± s.e, and n is the number of oocytes used.

Statistical analysis was performed by one-way ANOVA with Scheffé's *post hoc* analysis (p < 0.05).

### Mutation V1016I enhanced F1534C-mediated resistance to pyrethroids

**Fig 3:**
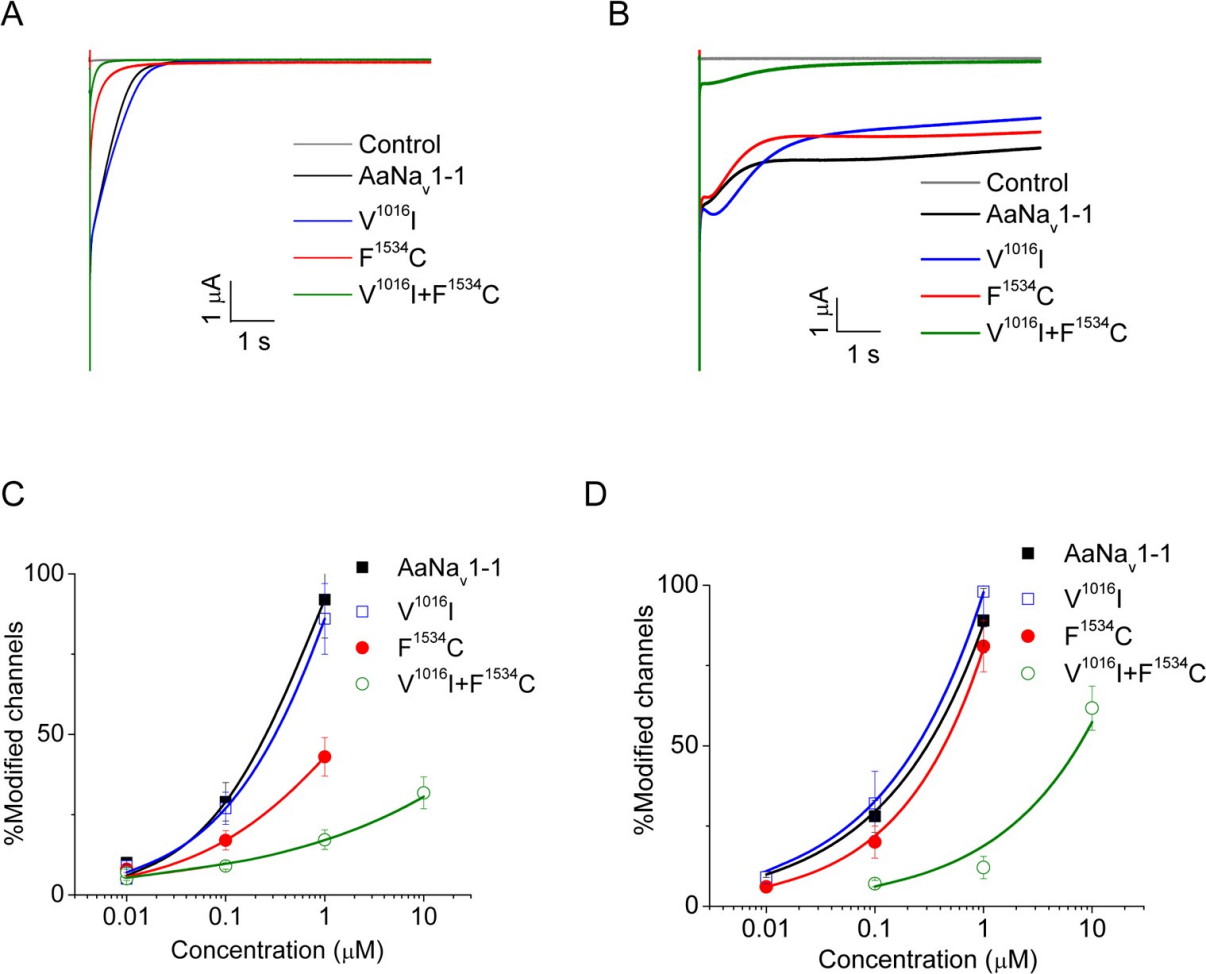
Effects of mutations V1016I and F1534C on the channel sensitivity to permethrin (PMT) and deltamethrin (DMT). (A) Representative tail currents induced by 1.0 μM PMT. (B) Representative tail currents induced by 1.0 μM DMT. (C) Dose-response curves of the channels modification by PMT. (D) Dose-response curves of the channels modification by DMT. The dose-response curve was fitting with Hill equation. Statistical significance was determined by using one-way ANOVA with Scheffé's *post hoc* analysis, and significant values were set at p < 0.05. The number of oocytes for each mutant construct was more than 6.

Mutations V1016I and F1534C were examined previously [32–34]. Here, we compared pyrethroid sensitivities of double mutant V^1016^I+F^1534^C and single mutation mutants V^1016^I and F^1534^C. We expressed the wild-type AaNa_v_1-1 and the mutants in *Xenopus* oocytes, measured tail currents induced by pyrethroids (Fig 3) using a multiple short-depolarizations protocol [39], and determined the percentage of channels modified by pyrethroids. The channel modification by pyrethroids increased in a dose-dependent manner (Fig 3). Consistent with earlier findings [32, 34], mutation F1534C conferred the channel resistance to PMT, but not to DMT (Fig 3), whereas mutation V1016I did not reduce the channel sensitivity to either PMT or DMT (Fig 3). However, V^1016^I+F^1534^C channel was not only more resistant to PMT than F^1534^C, but also showed resistance to DMT (Fig 3). Thus, although V1016I alone had no effect on the channel sensitivity to PMT or DMT, it enhanced the F1534C-mediated resistance to both PMT and DMT.

### Mutation T1520I enhanced F1534C-mediated resistance to Type I, but not to Type II pyrethroids

**Fig 4:**
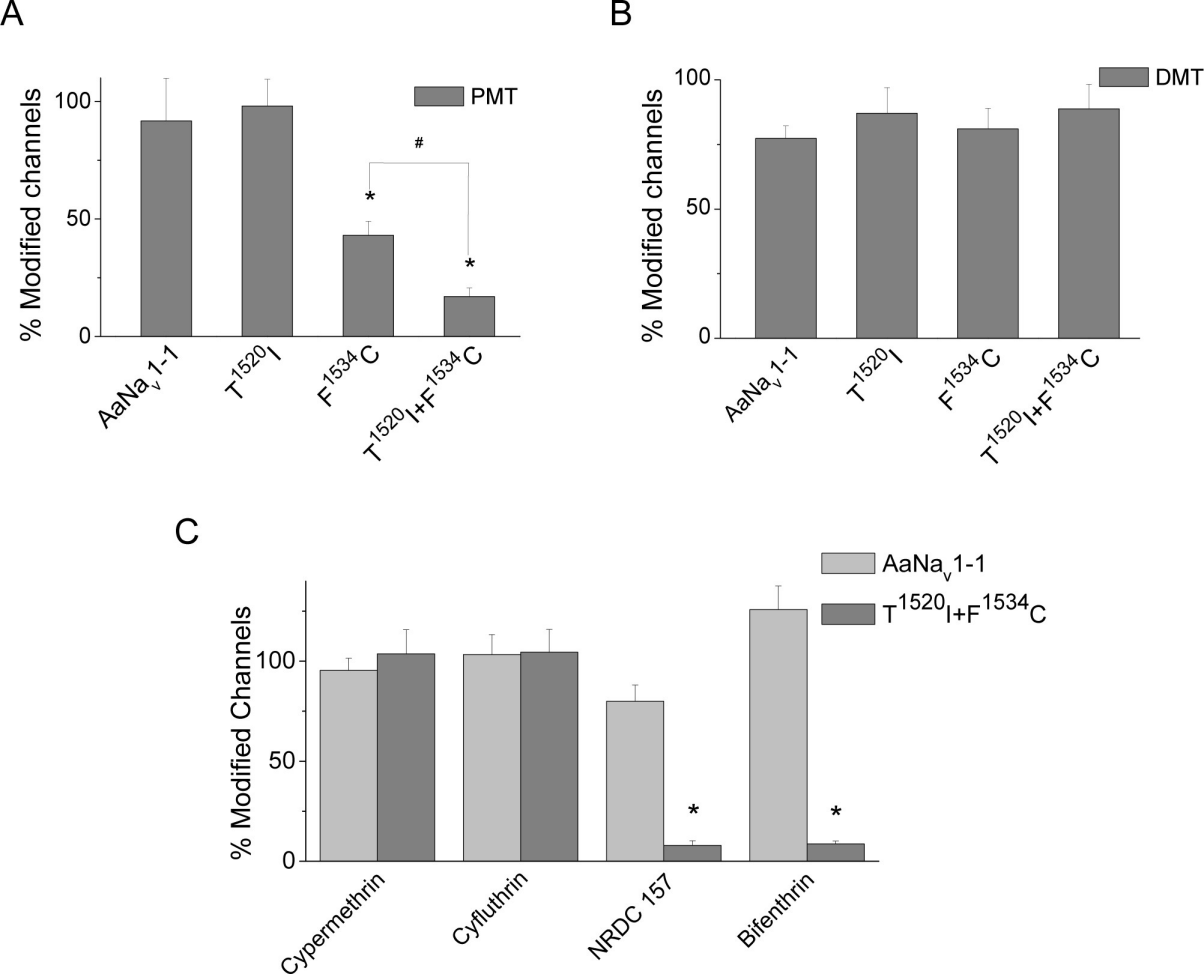
Effects of mutations T1520I and F1534C on the channel sensitivity to pyrethroids. (A) Channel modification by 1.0 μM PMT. (B) Channel modification by 1.0 μM DMT. (C) Channel modification by 1.0 μM cypermethrin, cyfluthrin, NRDC 157 and bifenthrin. The number of oocytes for each mutant was > 6. The asterisks indicate significant differences from the AaNa_v_1-1 channel as determined by using the one way ANOVA with Scheffé's *post hoc* analysis (p < 0.05). The pound sign indicates a significant difference in sensitivity to PMT between mutants as determined using one-way ANOVA with Scheffé's *post hoc* analysis (p < 0.05).

To evaluate the role of T1520I in pyrethroid resistance, we introduced T1520I alone or together with F1534C. Like V1016I, T1520I alone did not reduce the channel sensitivity to PMT or DMT (Fig 4A and 4B). However, T^1520^I+F^1534^C channel showed greater resistance to PMT, compared to that of F^1534^C channel (Fig 4A), but were not resistant to DMT. Thus, unlike V1016I, T1520I enhanced the resistance of F^1534^C channel to PMT, but not to DMT.

Previously the F^1534^C channel was found resistant to Type I, but not Type II pyrethroids [26, 28]. We further explored resistance of the double mutant channel T^1520^I+F^1534^C, to two Type I pyrethroids (bifenthrin and NRDC 157) and Type II pyrethroids (cypermethrin and β-cyfluthrin). β-cyfluthrin, cypermethrin and bifenthrin are commonly used in mosquito control. Consistent with data on PMT and DMT, T^1520^I+F^1534^C channel was resistant to Type I, but not Type II pyrethroids.

### Mutation V1016I, but not T1520I, conferred the AaNa_v_1-1 channel resistance to DDT

**Fig 5:**
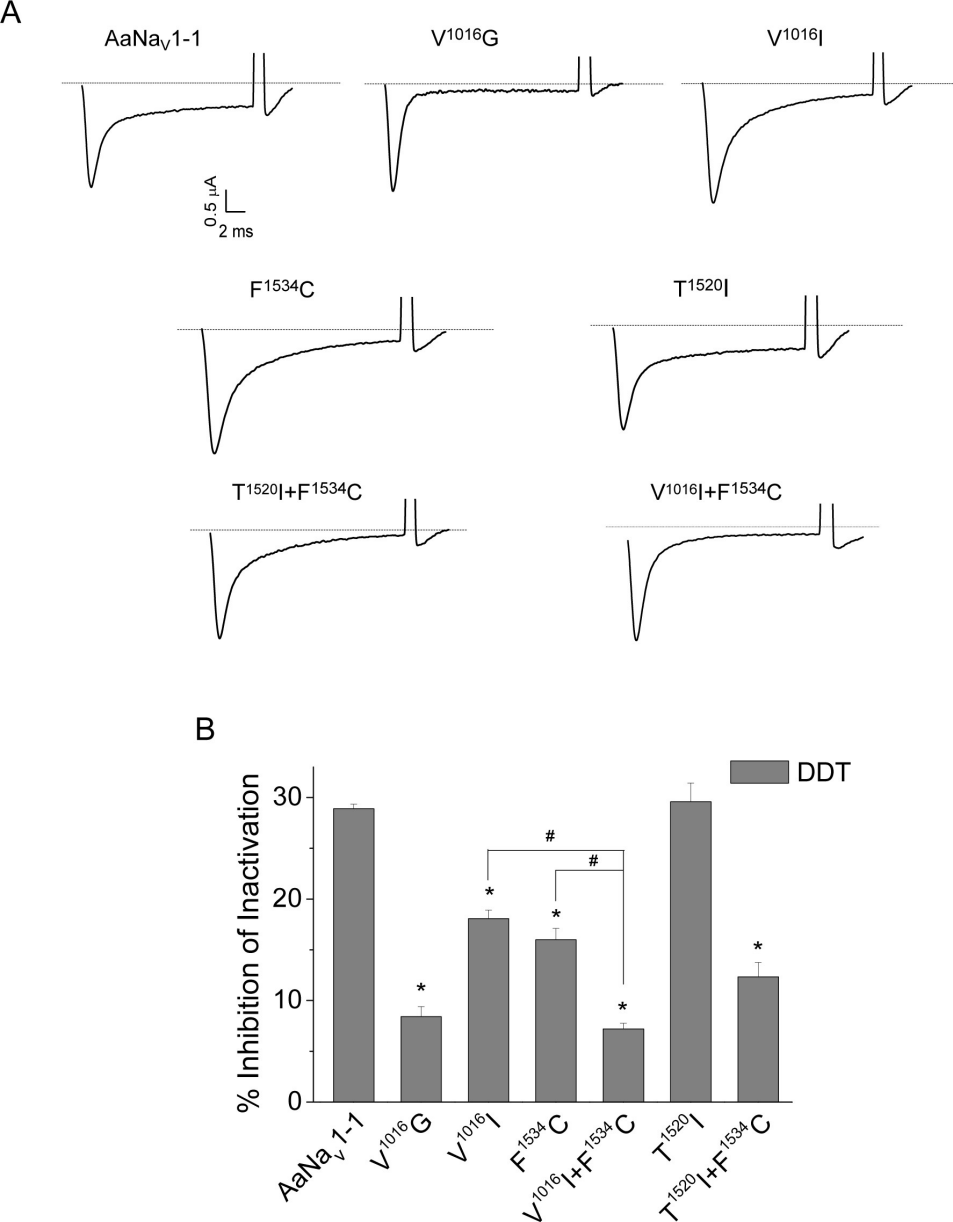
Effects of mutations on the channel sensitivity to DDT. (A) Representative traces from AaNa_v_1-1, V^1016^G, V^1016^I, T^1520^I, F^1534^C, T^1520^I+F^1534^C and V^1016^I+F^1534^C channels after incubation with DDT (100 μM). (B) Percentages of channel inactivation inhibited by DDT (100 μM). The number of oocytes for each mutant was more than 8. Error bars indicate mean ± s.e. The asterisks indicate significant differences in sensitivity of mutants versus wildtype to DDT as determined by using one-way ANOVA with Scheffé's *post hoc* analysis (p < 0.05). The pound sign indicates a significant difference in sensitivity to DDT between mutants as determined using one-way ANOVA with Scheffé's *post hoc* analysis (p < 0.05).

Although structurally distinct from pyrethroids, DDT also inhibits inactivation and deactivation of sodium channels [42, 43] and shares with pyrethroids two binding sites in insect sodium channels [32]. DDT induces extremely small and fast decaying tail currents in sodium channels and inhibits fast inactivation [41]. Therefore, we assessed the AaNa_v_1-1 sensitivity to DDT by measuring DDT-induced non-inactivating current. Fig 5A shows representative current traces from the AaNa_v_1-1 and mutants V^1016^G, V^1016^I, F^1534^C, T^1520^I, V^1016^I+F^1534^C and T^1520^I+F^1534^C. DDT caused smaller inhibition of fast inactivation in V^1016^G and V^1016^I than in the wild-type AaNa_v_1-1, indicating that the mutants were resistant to DDT. The V^1016^G channel was more resistant to DDT than the V^1016^I channel. In contrast, the T^1520^I channel was not resistant to DDT (Fig 5).

Previously we have shown that F1534C slightly reduces sensitivity of AaNa_v_1-1 to DDT [41]. Here we compared the DDT sensitivity of double mutants of T^1520^I+F^1534^C and V^1016^I+F^1534^C with that of F^1534^C. Channel T^1520^I+F^1534^C was more resistant to DDT than the wild-type channel, but there was no difference between the T^1520^I+F^1534^C and F^1534^C channels. However, the double mutant V^1016^I+F^1534^C conferred 2-fold more resistance to DDT than V^1016^I or F^1534^C channels.

### Residues V1016 and F1534, but not T1520 contribute to the pyrethroid receptor site PyR1 in the II/III repeat interface

**Fig 6:**
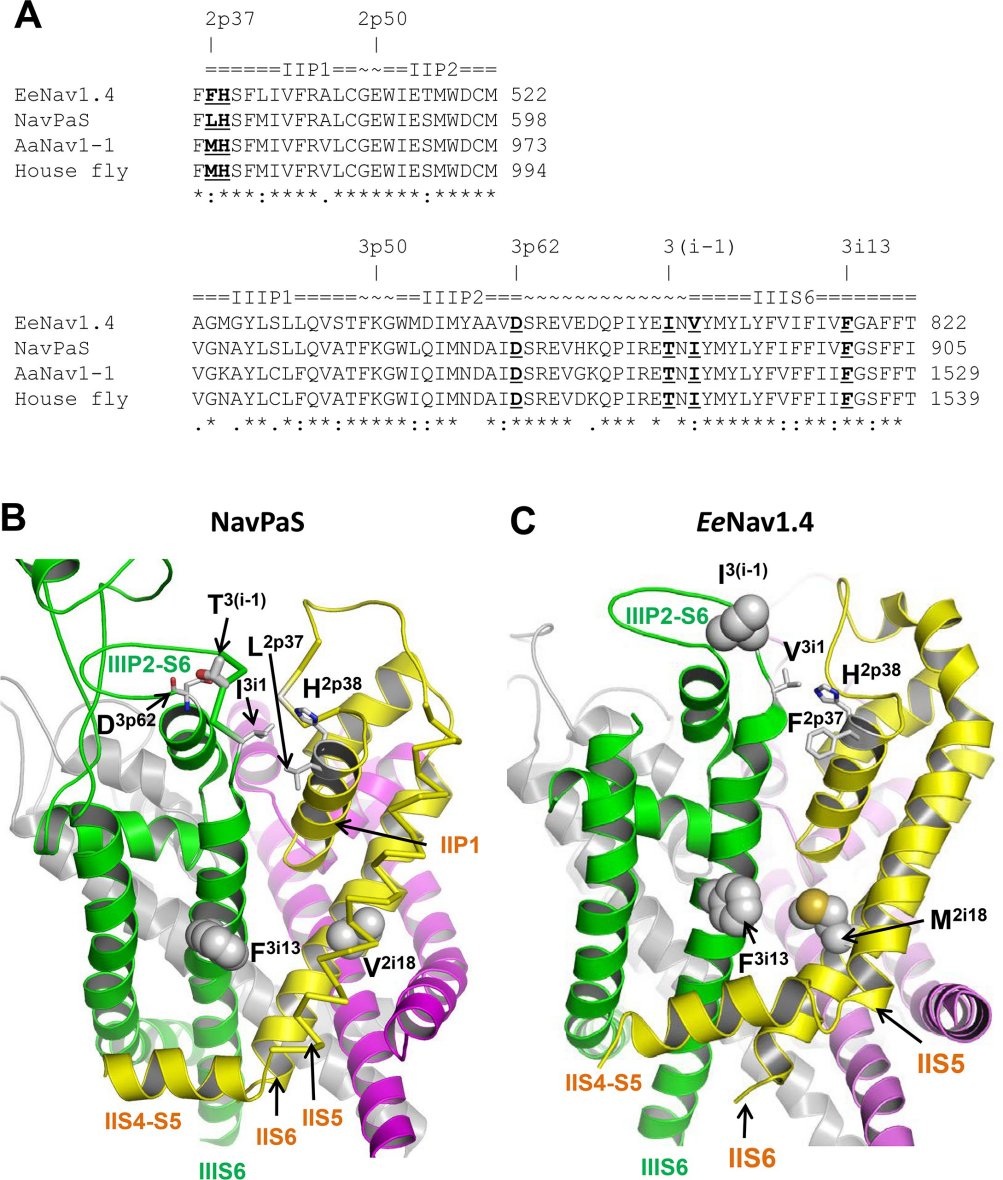
(A) Sequence alignment of sodium channel segments involved in the proposed mechanism by which mutation T^1520/3i(-1)^I allosterically induces small changes in the PyR1 site geometry. Highlighted are residues (except position 2i18), which are shown in panels B and C. Residue numbers in NavPaS and *Ee*Nav1.4 are sequential numbers in the PDB files of the cryo-EM structures where some segments are lacking. (B) and (C), Cryo-EM structures of eukaryotic sodium channels NavPaS (B) and EeNav1.4 (C). The pore-module helices in repeats I, II, III, and IV are magenta, yellow, green and gray, respectively. Side chains in positions 2i18 and 3i13, which correspond to AaNa_v_1-1 residues 1016 and 1534, are space-filled. In both channels, these residues are in the II/III repeat interface that harbors the pyrethroid receptor site PyR1. Threonine T^3(i-1)^ in the extracellular loop of NavPaS, which correspond to T^1520/3(i-1)^ in AaNa_v_1-1, is close to the N-terminus of IIIS6 and in AaNa_v_1-1 it cannot directly interact with PyR1-bound pyrethroids.

To describe homologous residues in various sodium channels we use labels, which are universal for P-loop channels [32, 44]. A label refers to the channel repeat (1–4), channel segment ("i" for the inner helix S6 and "p" for the P-loop), and relative position of the residue in the segment (Fig 6A). For example, following this nomenclature V1016 becomes 2i18. To facilitate recognition of residues in *Ae*. *aegypti*, we use both residues numbers and labels.

In homology models of AaNa_v_1-1, which are based on the X-ray structures of open eukaryotic potassium channel Kv1.2 [45] or open prokaryotic sodium channel NavMs [46], residues V^1016/2i18^ and F^1534/3i13^ are located, respectively, in helices IIS6 and IIIS6 and contribute to the pyrethroid receptor site PyR1 in the II/III repeat interface [26].

The cryo-EM structures of cockroach sodium channel NavPaS [47] and electric eel sodium channel Nav1.4 [48] confirm that residues in positions 2i18 and 3i13 are located in the II/III repeat interface (Fig 6B and 6C). F^3i13^ and M^2i18^ in *Ee*Nav1.4 (Fig 6C) are closer to each other than F^3i13^ and V^2i18^ in NavPaS (Fig 6B). It should be noted that IIS6 in the non-functional NavPaS is distorted likely due to a π-helix bulge above I^2i18^. Such bulges are not seen in IIS6 of *Ee*Nav1.4 or in the inner helices of prokaryotic sodium channels.

In NavPaS, T^3(i-1)^ (i.e., T1520) in the extracellular loop IIIP2-S6 is far from F^3i13^ and V^2i18^ and cannot directly interact with pyrethroids bound to the latter residues. In NavPaS, T^3(i-1)^ donates an H-bond to the backbone carbonyl of D^3p62^ in the AID motif at the C-terminus of helix IIIP2 (Fig 6B). This H-bond stabilizes the loop conformation. Channel AaNa_v_1-1 has the same-length loop IIIP2-S6 and the same AID motif (Fig 6A). Mutation T^3(i-1)^I eliminates the H-bond, thus destabilizing the loop conformation. The loop contains isoleucine I^3i1^, which forms tight inter-repeat contacts with residues L^2p37^ and H^2p38^ in the P-loop helix IIP1. AaNa_v_1-1 has the same residues I^3i1^, L^2p37^ and H^2p38^ (Fig 6A), which are likely involved in the inter-repeat contact. The IIIP2-S6 loop destabilization due to mutation T^3(i-1)^I would cause small shifts of IIIS6, IIP1, as well as IIS5 and IIS6, which form tight contacts with IIP1. Possible consequences of this changes are discussed in a later section.

## Discussion

Mutations V1016I and T1520I alone with F1534C cause high levels of pyrethroid resistance in *Ae*. *aegypti* field populations [13, 17, 49], but their effects on the sodium channel function and sensitivity to pyrethroids were unknown. Here we demonstrated that although neither mutation alone conferred pyrethroid resistance of mosquito sodium channels, they enhanced pyrethroid resistance caused by a common *Ae*. *aegypti* kdr mutation, F1534C. Specifically, V1016I+F1534C caused a high resistance to both a Type I pyrethroid, PMT, and a Type II pyrethroid, DMT, whereas T1520I+F1534C increased resistance to PMT, but not to DMT.

Our electrophysiological analysis in *Xenopus* oocytes established that F1534C confers sodium channel resistance only to Type I pyrethroids, but not to Type II pyrethroids including DMT, cypermethrin and cyfluthrin [32, 34]. These results are consistent with bioassay results from several field *Ae*. *aegypti* populations carrying the F1534C mutation [21, 50, 51]. For example, a population (Kota Bharu) from Malaysia was resistant to PMT, but not to DMT [21]. Populations with the homozygous F1534C mutation were susceptible to DMT [50] and λ-cyhalothrin, another Type II pyrethroid [51]. However, some field populations carrying the F1534C mutation were found to be resistant to DMT [52]. Similarly, although we show that the T^1520^I+F^1534^C channel is not resistant to Type II pyrethroids, populations in which T1520I+F1534C were detected were also resistant to DMT [17]. It is possible that in these populations of *Ae*. *aegypti* additional kdr mutation(s) in the sodium channel or other pyrethroid-resistance mechanisms, such as enhanced metabolic detoxification, contribute to resistance to DMT.

**Fig 7:**
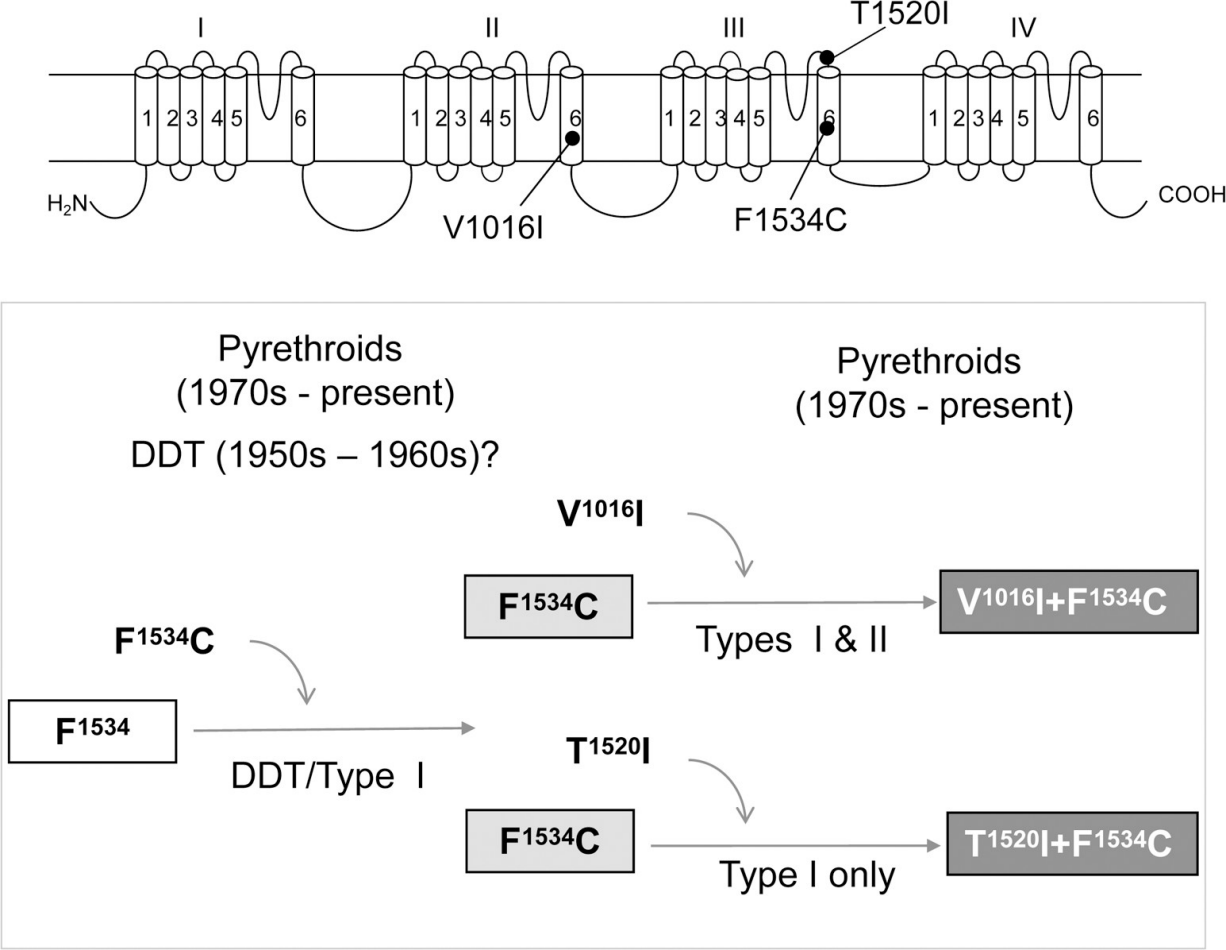
Sequential selection of kdr mutations for pyrethroid resistance in *Ae*. *aegypti*. Darker background colors indicate higher levels of resistance. V1016 and T1520I are selected in mosquitoes carrying F1534C. F1534C (and probably V1016I) emerged under the DDT pressure prior to usage of pyrethroids. T1520I was selected under pressure of Type I pyrethroids.

Since V1016I or T1520I alone do not confer any resistance to pyrethroids, we suggest that likely they have been selected in populations with the background kdr mutation F1534C established in the field (Fig 7). Our results support the hypothesis on sequential evolution of F1534C and V1016I proposed in 2015 by Vera-Maloof et al. [49]. The hypothesis was based on a linkage disequilibrium analysis of the two mutations in *Ae*. *aegypti* collected in Mexico from 2000 to 2012. It is also consistent with the observation that in natural populations frequencies of F1534C are higher and increase more rapidly than frequencies of V1016I [14, 49, 53]. V1016I likely has emerged in the F1534C background in response to intensive use of pyrethroids in mosquito control. Interestingly, while the F^1534^C channel is resistant to only Type I pyrethroids, V^1016^I+F^1534^C is highly resistant to both Type I and Type II pyrethroids. Thus, V1016I enhanced F1534C-mediated pyrethroid resistance to both Type I and Type II pyrethroids. We suggest a similar evolution path (Fig 7) for the emergence of T1520I in the background of *Ae*. *aegypti* populations carrying F1534C in India [17].

Another mutation, V410L in IS6, was recently found to co-occur with F1534C and V1016I in pyrethroid resistant populations in Brazil [10], Mexico [54] and Colombia [51]. Unlike V1016I and T1520I, V410L alone confers resistance to both Type I and Type II pyrethroids. V410L could be selected independent of F1534C. However, while the frequency of the triple mutation increased drastically from 2000 to 2006, heterozygote/homozygotes of V410L or V1016I without F1534C were detected at extremely low frequencies [54]. These results suggest that in these populations, strong selection pressure favors the haplotype carrying all three mutations, which likely confer the greatest level of pyrethroid resistance. Whether the concurrence of V410L+V1016I+F1534C provides fitness advantages remains to be determined.

The pyrethroid resistance augmentation caused by mutations V1016I and T1520I in *Ae*. *aegypti* is reminiscent to that of kdr mutations in *Anopheles gambiae* mosquitoes and German cockroach *Blattella germanica* [35, 55, 56]. For example, mutation N1575Y detected in pyrethroid-resistant *An*. *gambiae* populations [57], has no effect on the action of pyrethroids, but enhances pyrethroid resistance caused by mutations L1014F/S/W [56]. In cockroaches, mutations E435K and C785R alone did not reduce sodium channel sensitivity to pyrethroids. However, concurrence of either E435K or C785R with other kdr mutations, V410M in IS6 or L1014F in IIS6, significantly increases pyrethroid resistance [42]. Collectively, these data reflect complexity of interactions of pyrethroids with sodium channels and indicate sequential evolution of insect resistance to pyrethroids.

It is unknown whether or not the *kdr* mutations explored in this study were selected in populations due to intensive use of DDT prior to introduction of pyrethroids in the 1970s. The first case of insect resistance to DDT was documented by Busvine in 1951 [58]. The resistance emerged after widespread house-spraying with insecticides including DDT during 1947–50 to eradicate Anopelines. Furthermore, one DDT resistant strain was also cross-resistant to pyrethrins, naturally occurring prototypes of pyrethroids [58]. Although the DDT resistance was detected in the field [59, 60], decades passed before the first evidence that resistance to DDT could be caused by the sodium channel *kdr* mutations, which confer resistance to pyrethroids. For example, V1016G and F1534C (Fig 5), as well as several other *kdr* mutations, such as L1014F, confer sodium channel resistance to DDT [61]. Therefore, it is possible that some *kdr* mutations, such as V1016G and F1534C, could have emerged due to intensive DDT use in eradication of malaria and other arthropod pest management programs in the 1950s and 1960s, before pyrethroids were introduced [62, 63]. It would be interesting to examine in future whether insect specimens collected in the 1950s and 1960s carry some *kdr* mutations. Some *kdr* mutations likely have appeared due to pyrethroid selection. For example, recently detected T1520I does not confer pyrethroid or DDT resistance by itself, nor does it increase F1534C-mediated resistance to DDT (Fig 7).

How mutation T^1520/3i(-1)^I may augment pyrethroid resistance of channel F^1534/3i13^C? In NavPaS T^1520/3i(-1)^ is far above F^1534^/^3i13^ (Fig 6B) and cannot directly interact with PyR1-bound pyrethroids or DDT, consistent with our data that point mutation T^1520/3i(-1)^I alone does not affect action of pyrethroids or DDT (Fig 4 and Fig 5). The fact that T^1520/3i(-1)^I increases the channel resistance to PMT, which is caused by F^1534/3i13^C (Fig 4), indicates that T^1520/3i(-1)^I allosterically induces a small deformation of PyR1.

Double mutant T^1520/3(i-1)^I+F^1534/3i13^C was much more resistant to PMT than F^1534/3i13^C, but remained sensitive to DMT (Fig 4). This concords with our data that point mutation F^1534/3i13^C decreases action of PMT, but not that of DMT (Fig 3). A possible cause is that DMT, but not PMT has a nitrile group, which is proposed to accept an H-bond from threonine T^2o10^ in IIS5 [64]. This H-bond would attract DMT closer to IIS5 and therefore shift farther from IIIS6, making the DMT action insensitive to mutation F^1534/3i13^C.

Substitution of the hydrophobic beta-branched V^1016/2i18^ with the hydrophobic beta-branched isoleucine has no impact the channel resistance to PMT or DMT (Fig 3). However, the fact that channel V^1016/2i18^I+F^1534/3i13^C is resistant to both DMT and PMT suggests that I^1016/2i18^, which is larger than V^1016/2i18^, shifts the PyR1-bound ligand closer to helix IIIS6 making action of pyrethroids sensitive to mutation in position i13, which is located against position 2i18 in the II/III repeat interface (Fig 6B and 6C).

In our Kv1.2-based model of mosquito sodium channel [41], DDT directly interacts with F^3i13^ in the PyR1 site and is less than 5 Å from V^2i18^, which also contributes to the PyR1 site. This model is consistent with our data that mutations V^1016/2i18^G/I, F^1534/3i13^C and T^1520/3(i-1)^I+F^1534/4i13^C increase the channel resistance to DDT (Fig 5). Furthermore, our data that point mutation T^1520/3(i-1)^I does not affect the channel resistance to DDT (Fig 5) is consistent with our proposition that this mutation induces a small change in the PyR1 site geometry. This change, however, increases the DDT resistance in the double mutant T^1520/3(i-1)^I+F^1534/3i13^C as it does in case of PMT. Thus, the experimental data of this study can be explained in view of our previous models of mosquito sodium channel and recent cryo-EM structures of eukaryotic sodium channels. Furthermore, the latter structures suggest an allosteric mechanism by which mutation T^1520/3(i-1)^I induces a small change of the PyR1 site geometry.

In conclusion, our functional characterization revealed common and unique effects of V1016I and T1520I mutations on the channel sensitivity to DDT and Type I and Type II pyrethroids. Our results supported the hypothesis on sequential selection of kdr mutations in *Ae*. *aegypti*: F1534C (and probably V1016I) appeared first in response to DDT, while T1520I emerged later under the continued pyrethroid selection. Both V1016I and T1520I can be established in kdr populations that possess the F1534C mutation.
